# The regulation, function, and role of lipophagy, a form of selective autophagy, in metabolic disorders

**DOI:** 10.1038/s41419-022-04593-3

**Published:** 2022-02-08

**Authors:** Sheng Zhang, Xueqiang Peng, Shuo Yang, Xinyu Li, Mingyao Huang, Shibo Wei, Jiaxing Liu, Guangpeng He, Hongyu Zheng, Liang Yang, Hangyu Li, Qing Fan

**Affiliations:** Department of General Surgery, The Fourth Affiliated Hospital, China Medical University, Shenyang, 110032 China

**Keywords:** Autophagy, Mechanisms of disease

## Abstract

Autophagy is a conserved method of quality control in which cytoplasmic contents are degraded via lysosomes. Lipophagy, a form of selective autophagy and a novel type of lipid metabolism, has recently received much attention. Lipophagy is defined as the autophagic degradation of intracellular lipid droplets (LDs). Although much remains unknown, lipophagy appears to play a significant role in many organisms, cell types, metabolic states, and diseases. It participates in the regulation of intracellular lipid storage, intracellular free lipid levels (e.g., fatty acids), and energy balance. However, it remains unclear how intracellular lipids regulate autophagy. Impaired lipophagy can cause cells to become sensitive to death stimuli and may be responsible for the onset of a variety of diseases, including nonalcoholic fatty liver disease and metabolic syndrome. Like autophagy, the role of lipophagy in cancer is poorly understood, although analysis of specific autophagy receptors has helped to expand the diversity of chemotherapeutic targets. These studies have stimulated increasing interest in the role of lipophagy in the pathogenesis and treatment of cancer and other human diseases.

## Introduction

Autophagy has been intensely studied since its discovery. As one of the two evolutionarily conserved cell degradation pathways (autophagic degradation and proteasomal degradation), autophagy maintains a dynamic intracellular balance and can be activated by a variety of different stresses [1–4]. Autophagy is divided into three subtypes (macroautophagy (herein referred to as autophagy), chaperone-mediated autophagy (CMA), and microautophagy), which can be nonselective or selective [5] and include the selective removal of damaged mitochondria by mitophagy and the selective removal of endoplasmic reticulum components by phagocytosis [3, 5–7]. Intracellular lipids are an indispensable source of energy for cells, the structural components of membranes, are synthesized to produce other types of molecules (e.g., hormones), and participate in cell signal transduction. Storing an appropriate amount of lipids to perform cellular functions when needed is essential to cell survival. In 2009, it was discovered that macroautophagy can selectively degrade lipids in hepatocytes; the authors then coined the term “lipophagy” to describe the process [8]. This discovery gave scientists a new perspective and understanding of how lipid metabolism regulates cellular physiology and pathology. Since then, many new functions of autophagic lipid metabolism have been gradually discovered. In this review, we mainly introduce some regulatory effects and functions of lipophagy, including regulating intracellular lipid storage to maintain energy supply under emergency conditions and promoting potential lipotoxic molecular metabolism. Then, we introduce a variety of metabolic disorders in which lipophagy may be involved, including fatty liver disease, and the role of lipophagy in cancer progression.

## What is lipophagy?

### Lipophagy: a novel type of lipid catabolism

Lipid metabolism is a complex metabolic process that includes digestion, absorption, synthesis, catabolism and peroxidation. As one of the most important sources of nutrition, lipids provide energy and essential fatty acids for the human body. Under stress conditions such as hunger and hypoxia, nutrient depletion requires the mobilization of free fatty acids to supply energy, which connects the functions of lipid metabolism, and autophagy. There are three types of lipids: triglycerides (TGs), steroids, and phospholipids [9]. The chemical name of TGs is triacylglycerol (TAG), also known as fat, which is the main lipid storage carrier and consists of one glycerol molecule and three fatty acids. Usually used for ingested food, adipogenesis, the process of TG synthesis, is mainly performed in the liver. TAG is generally stored in lipid droplets (LDs) and is hydrolyzed and metabolized according to the different levels of fat in different parts of the body.

Changes in lipid storage are involved a variety of different pathological conditions, including obesity, inflammation, fatty liver, atherosclerosis, neurodegenerative diseases, and cancer. Excessive lipid accumulation can lead to hypoxia, endoplasmic reticulum stress, high immune cell infiltration, increased secretion of proinflammatory cytokines and the development of obesity. Insufficient lipid storage or malnutrition lead to lipotoxicity, mitochondrial dysfunction, increased oxidative stress, and other consequences. Therefore, maintaining proper lipid content is very important for the normal operation of the human body [10].

**Fig. 1:**
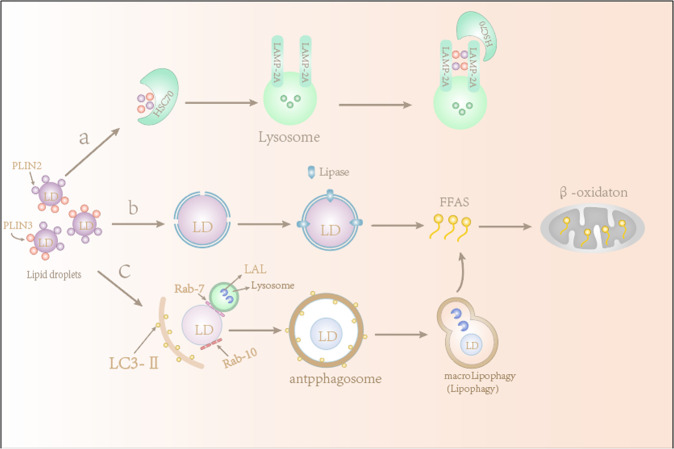
The lipolysis and lipophagy pathways of lipid droplets. **a** Chaperone-mediated autophagy degrades the LD coat proteins perilipin 2 and perilipin 3 through the coordinated action of heat-shock cognate 71 kDa protein (HSC70) and receptor lysosome-associated membrane glycoprotein 2A (LAMP2A). **b** Degradation of the LD coat proteins PLP2 and PLP3 allows lipase- and fat-soluble organelles to enter and undergo lipolysis. **c** Lipophagy induces engulfment of small cytoplasmic lipid droplets or large cytoplasmic lipid droplets by LC3-II-positive membranes. Lipid droplets are transported to lysosomes and degraded by lysosomal acid lipase (LAL). Rab7 promotes the decomposition of LDs by interacting with its downstream effector RILP. The fatty acids produced are mainly directed to the mitochondria to participate in the β-oxidation pathway.

There are two ways that cells can metabolize stored fats: lipolysis and lipophagy [8, 11]. Once LDs are converted to free fatty acids, they are degraded through mitochondrial fatty acid β-oxidation to meet the energy needs of rapidly proliferating cells [12] (Fig. 1). Lipid metabolism is involved in regulating a variety of cellular processes, including autophagy and the formation of autophagy-related membrane structures. The transfer and degradation of LDs containing TAG through the lysosomal pathway are mediated by autophagy, a process that is now called lipophagy. Lipophagy was originally described in hepatocytes because hepatocytes can accumulate excess lipids. Researchers found that in ATG7 knockout mice, the LD decomposition in mouse hepatocytes depends on autophagy [8].

It is not surprising that lipid levels affect autophagy levels. Under an electron microscope [13], the number of autophagosomes and the protein level of autophagy factors was decreased in mice with long-term hyperlipidaemia and hereditary obesity. Another study showed that the number of autophagosomes in the liver of obese mice was increased, the fusion of autophagosomes and lysosomes was normal, and autophagy defects were caused by a decrease in cathepsin L and lysosomal acidification defects, which prevented the substrate degradation of autophagic lysosomes [14]. Some studies attempting to assess in vitro autophagosome–lysosome fusion have also reported liver organelle fusion defects in mice fed a high-fat diet [15], which were due to changes in membrane lipid composition caused by the high-fat diet. Defects in the formation and fusion of autophagosomes and the level of substrate degradation may be the mechanism underlying liver autophagy-related injury. Therefore, studying the effect of chronic lipid overload on liver autophagy may be important for the treatment of related diseases. Evidence of lipophagy has been found in models other than HFD-fed mice. It has been shown that 1,3-dichloro-2-propanol can induce lipid accumulation in HepG2 cells by reducing autophagosome formation and impairing lysosomal formation via AKT/mTOR [16]. Several studies have found that a deficiency in selective autophagy is associated with suppression of lipid oxidation. The loss of ATG7 or ATG5 in the liver significantly reduces the production of ketone bodies during hunger or fasting due to the interaction between nuclear receptor coinhibitor 1 (NCoR1) and PPARα; this effect inhibits PPARα transactivation and causes PPARα binding to GABARAP proteins to be degraded by autophagy. This illustrates the role of autophagy in regulating β-oxidation and ketone body production [17].

From another point of view, there may be an opposite relationship between autophagy and lipolysis. Neutral lipase-mediated LD destruction regulates the biogenesis and autophagy of autophagosomes. In HeLa cells, low-density lipoprotein is necessary for autophagy and dynamically interacts with early autophagosomes [18]. The enhancement of autophagy mediated by LDs depends on PNPLA5 and other lipid biosynthesis and remodeling enzymes. PNPLA5 is a neutral triglyceride lipase. As a lipid intermediate for LDs and TGs that promotes autophagy membrane biogenesis, PNPLA5 is involved in autophagy ligand degradation, mitochondrial quality control and microbial clearance [18]. This suggests that lipids mobilized by neutral lipases from LDs provide a key lipid building block for autophagy membranes. However, autophagy is needed in LDs not only to provide structural lipids but also to meet energy requirements or induce lipid-related effects in signaling pathways.

Yeast studies [19] have provides us with more evidence of the relationship between LDs and autophagosomes. Under nitrogen starvation, chemical or genetic inhibition of free fatty acid (FFA) synthesis prevents the formation of autophagosomes, as determined by Atg8, which can be restored by adding FFAs. However, in yeast with LD deficiency, similar autophagy defects cannot be reversed by FFAs, indicating that LDs alone, and not the decomposition of FFAs, are important. Studies of different yeast mutants have shown that autophagy membrane formation requires the degradation of TGs and steroid esters stored in LDs that are supplied to the endoplasmic reticulum [18] through direct contact between two organelles. However, other studies have shown that although the autophagy function of yeast lacking LDs is impaired under starvation, the autophagy mechanism is not affected because rapamycin can still induce autophagy [20] in the absence of LDs. These changes in phospholipid composition and free fatty acids lead to endoplasmic reticulum stress and changes in endoplasmic reticulum lipid composition, resulting in autophagy defects. Whether these findings indicate that blocking the lipophagy pathway does not affect autophagy or whether they prove the function of lipolysis in regulating autophagy still needs to be determined. However, these perspectives describe the complex interaction between lipid metabolism and autophagy, reveal a novel connection between lipid metabolism and lipophagy, aid the identification of selective receptors for lipophagy, and increase the understanding of the interactions between lipid metabolism and autophagy.

### Lipophagy-targeting LDs for selective autophagy

**Fig. 2:**
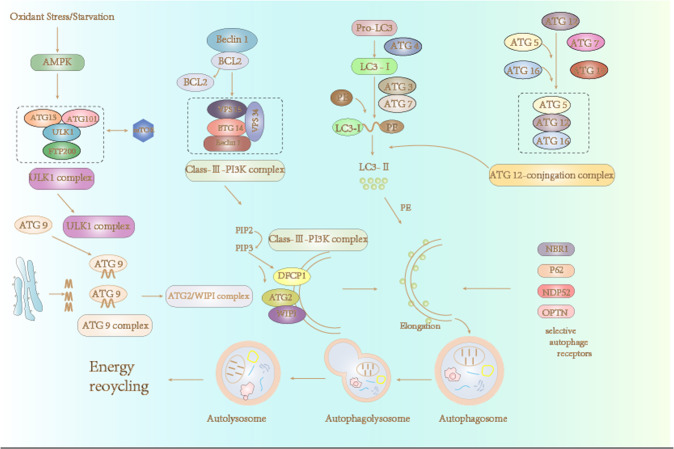
Autophagy in mammalian cells. The initiation of autophagy is dependent on six distinct protein complexes formed by the following proteins: ULK1, class III PI3K (phosphoinositide 3-kinase), ATG9, ATG2-ATG18, ATG8/LC3, and ATG12.

Autophagy, as a catabolic pathway induced by the strict regulation of cell homeostasis and stress in eukaryotes, is comprised of three subtypes: macroautophagy, microautophagy, and CMA. Although they all result in lysosomal or protein degradation as a means of quality control, they are different in mechanism [21, 22]. Microautophagy is the most atypical of the autophagic pathways and involves phagocytosis and the degradation of cytoplasmic contents through invasion of the lysosomal membrane [23, 24]. Macroautophagy is the most widely studied type of autophagy; it occurs more frequently and requires dozens of different autophagy-related proteins to coordinate the delivery of cytoplasmic content to the lysosome. In macroautophagy, double-membrane autophagosomes are formed under hunger or stress, which isolate the contents and aid in their transportation to the lysosome [25] (Fig. 2). CMA degrades a single soluble protein with specific amino acid characteristics (KFERQ motif), which is recognized by cytoplasmic heat shock-associated protein 70 (Hsc70) and connected to the lysosome by receptor lysosome-associated membrane protein (LAMP2A) [21].

Studies over the past decade have shown that although autophagy was initially thought to be a large-scale degradation pathway stimulated by cellular stressors, including starvation, autophagy can also selectively identify specific types of cargo for degradation [26, 27]. Selective autophagy can occur continuously to maintain intracellular homeostasis, and specific stimuli can activate the necessary selective autophagy pathway to manage specific stressors [28]. The results of dysregulation of selective autophagy reveal its importance in maintaining intracellular homeostasis, and selective autophagy plays a role in a variety of disease pathologies, including atherosclerosis, nerve deformation, and cancer [19, 29–32]. The naming of specific selective autophagy pathways is often dependent on the substrate that is degraded. For instance, the process for the selective removal of mitochondria is called mitophagy, selective removal of ferritin is called ferritinophagy, selective removal of the ER is called reticulophagy, and selective removal of ribosomes is called ribophagy [18, 26, 33–37]. Lipophagy is a form of autophagy that selectively engulfs cellular LDs and has been shown to occur in a variety of cells, including neurons, brown adipose tissue cells, renal tubular cells, Leydig cells, enterocytes and foam macrophages [38–43]. There are two forms of lipophagy: chaperone-mediated lipophagy and macrolipophagy (herein referred to as lipophagy).

To date, studies have shown that the important proteins and pathways that maintain lipid phagocytosis and lipid droplet homeostasis include RabGTP enzymes located in LDs, Rab10 and Rab7 (mainly activated under autophagy), and the rapamycin complex (MTORC1)-periliptin-3 pathway. During lipophagy, the phosphorylation of perilipins by PKA and its degradation by the proteasome occur first. Second, perilipins are recognized by the heat-shock cognate protein HSPA8/Hsc70 and bind the lysosomal membrane receptor LAMPA2; they are then localized to the lysosome [44–46] in a process termed CMA [44]. A cascade of lipases and esterases is triggered by the degradation of perilipins, which produce fatty acids, glycerol, and cholesterol [47] that interact with Patatin-like phospholipase domain-containing 2 (PNPLA2) to undergo lipolysis. PNPLA2 is a calcium-independent phospholipase, also known as an adipocyte triglyceride lipase. It can interact with LC3b to attach LDs to the autophagosome membrane [48, 49] or interact with autophagy cargo receptor isolator-1/p62 to recruit LDs [50]. Lipolysis can activate sirtuin1 via allosteric activation of monounsaturated lipids produced from LDs [51]. Sirtuin1 can then stimulate mitochondrial biogenesis and help maintain lysosomal function by activating the receptor γ costimulatory factor PGC1α [52, 53]. Notably, perilipins can connect lipids stored in LDs and transfer them to other organelles, functioning in lipid-mediated communication between the organelles [54, 55]. If dysfunction occurs, these lysosomes produce secretions containing unique lipids and protein hydrolysates that can be degraded in the extracellular matrix via lysosome exocytosis, which can lead to inflammation [56] as well as tumor invasion and metastasis [57].

Rab7 regulates the transport of lysosomes and multivesicular bodies to LDs [58] (Fig. 1). Rab10 recruits LC3-positive autophagy membranes to LDs and promotes the phagocytosis of LDs into autophagosomes by binding to adaptor protein EH domain binding protein 1 (EHBP1) and membrane deformed ATP EH domain-containing 2 (EHD2) to form a complex [59], which is essential to lipophagy in hepatocytes. The selective autophagy of organelles involves specific receptors that initiate autophagy around the organelle by binding to LC3. Autophagic recruitment is mainly mediated by organelle-resident receptors such as Atg32, Nix, and BNIP, which contain Atg8 family interaction motifs or LC3 interaction regions; it is also mediated by LIR-containing adapter proteins such as OPTN, NBR1, TAX1B1, p62, and NDP52, which bind to ubiquitinated cargo [60]. It is not clear whether lipophagy requires specific receptors or other proteins to recruit autophagic membranes, but many studies have suggested that p62 may act as a lipophagy receptor because p62 is necessary for lipophagy and is associated with LDs [15, 50, 61–63]. As a scaffold protein, p62 has been widely studied in a variety of signal transduction pathways, many of which are related to cell survival and death [64–67]. To date, ubiquitination appears unrelated to p62 recruitment and lipophagy of LDs. Which mechanism mediates autophagy targeting LDs, whether ubiquitin is involved in the targeting process, and whether there are other lipophagy-specific receptors remain to be determined.

## Regulation and function of lipophagy

### The size of lipid droplets affects the level of lipophagy

LDs are a type of neutral lipid storage organelle that originates from the ER [68], which is composed of a neutral lipid hydrophobic core and an external phospholipid monolayer [69] and is the hub of cellular lipid and energy metabolism [70–72]. The outer layer of the ER contains resident and temporary membrane proteins that regulate the transport of LDs and contact with other cell organelles for lipid transfer [73, 74]. The initiation of lipophagy, as well as lipase access to the storage core of mainly neutral acylglycerides and cholesterol esters, is mainly regulated by perilipins [75]. In contrast to the ER and other organelles, lipid droplet membranes are mainly composed of phosphatidylcholine, followed by phosphatidylethanolamines, phosphatidylinositols, phosphatidylserines, and sphingomyelins, as well as a small amount of free cholesterol and phosphatidic acids [76].

As a dynamic source of stored lipids, LDs can be rapidly mobilized to release fatty acids that can be either degraded into energy via β-oxidation, used in membrane synthesis, and/or used as lipid signaling molecules [71]. LDs modulate cell survival [77], ER stress [78], and mitochondrial dysfunction caused by free fatty acids [79, 80] by isolating lipids and inhibiting lipotoxicity.

Fatty acids in the core of LDs, in the form of triglycerides, can be mobilized by lipolysis or lipophagy [71] to participate in metabolic processes and membrane biosynthesis during the cell growth phase of membrane expansion and high phospholipid biosynthesis. Free fatty acids can affect membrane homeostasis and disrupt membrane integrity [81]; capturing fatty acids in LDs can prevent the potentially deleterious activities of fatty acids and their derivatives [82, 83]. Thus, once storage of fatty acids in LDs is impaired or overloaded, it may lead to lipotoxicity, which can cause diseases such as nonalcoholic fatty liver disease (NAFLD) and type 2 diabetes [13, 84].

Similarly, in tumor cells, continuously activated lipid synthesis and uptake have been found to play an important role in tumor cell resistance to “hunger”. Excess lipids will be stored in LDs, initiate decomposition and provide energy during “hunger”. For example, in hepatocellular carcinoma cells, the metabolic enzyme PCK1 (cytosolic phosphoenolpyruvate carboxykinase 1) is endowed with a nonmetabolic enzyme function, which promotes the activation of the SREBP (sterol regulatory element-binding proteins) signaling pathway and enhances lipid droplet synthesis in tumor cells [38]. How are many LDs degraded to deal with hunger; how are lipolysis and lipophagy, as two possible metabolic pathways of lipid metabolism, targeted by LDs; do these two pathways crosstalk with each other; do these two pathways exist in series or in parallel; and are these two pathways related to the properties of LDs? There is now more evidence to answer such questions. After continuous exploration in recent years, researchers have found that the size of LDs is probably the most important factor affecting and restricting the regulation of lipophagy and lipolysis.

**Fig. 3:**
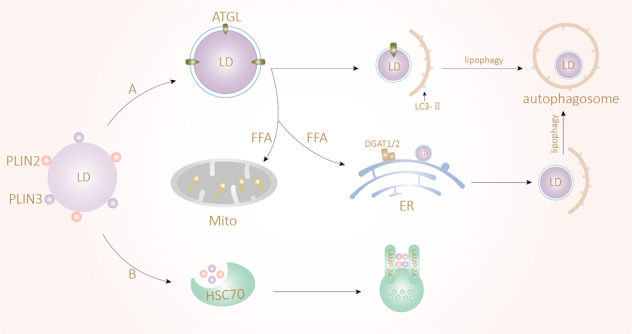
Lipolysis and lipophagy may be sequential pathways. **A** Larger lipid droplets cannot be degraded by lipophagy and need to be degraded by ATGL-driven lipolysis. Lipolysis can reduce the size of lipid droplets. Some LDs escape β-oxidation in the endoplasmic reticulum and become esterified into small lipid droplets by DGAT1/2, at which point they are degraded via lipophagy. Lipolysis and lipophagy may be sequential pathways. **B** Synergistic degradation of the lipid droplet coat proteins PLIN2 and PLIN3 occurs via heat-shock protein (HSC70) and receptor lysosome-associated membrane glycoprotein 2A (LAMP2A).

In fact, researchers have consistently found that the content of LDs is affected by the inhibition of lipophagy or lipolysis pathways. In in vivo and in vitro hepatocyte experiments, researchers found that ATGL (a rate-limiting cytoplasmic lipase) preferentially acted on the largest LDs in hepatocytes, while lipid phagocytosis affected only small cytoplasmic LDs <1 mm in diameter. Inhibition of ATGL leads to the accumulation of large LDs; furthermore, knockout of LAL leads to accumulation of many small LDs in a manner dependent on lipolysis driven by upstream ATGL, and inhibition of ATGL affects the shape and size of LDs more rapidly. Activating the autophagy lysosome pathway alone cannot completely remove excess lipids; in the case of combined inhibition, only the accumulation of large LDs is caused [85]. These experimental results suggest that lipolysis and lipophagy may be tandem pathways, with lipolysis mainly targeting large LDs to produce small LDs that can be degraded by lipophagy and playing a role upstream of the lipophagy pathway (Fig. 3). We think that lipophagy is an alternative way by which traditional lipase can drive lipid droplet degradation or a novel pathway of lipid catabolism. In another experiment, the researchers found that in liver transplantation models, mutations in PNPLA3 caused larger LDs to accumulate in the liver and accelerated fibrosis. Older donors had more large LDs, which may be due to the decrease in mitochondrial β-oxidation caused by hepatocyte senescence, while small LDs may induce decomposition of LDs [86] by activating the lipophagy pathway. In recent years, several experiments have gradually developed methods to change the size of fat droplets to treat obesity and fatty liver [87, 88]. However, whether it is better to target lipolysis or lipophagy or regulation of both requires further experimental exploration.

### Lipophagy mediates resistance to cell death

**Fig. 4:**
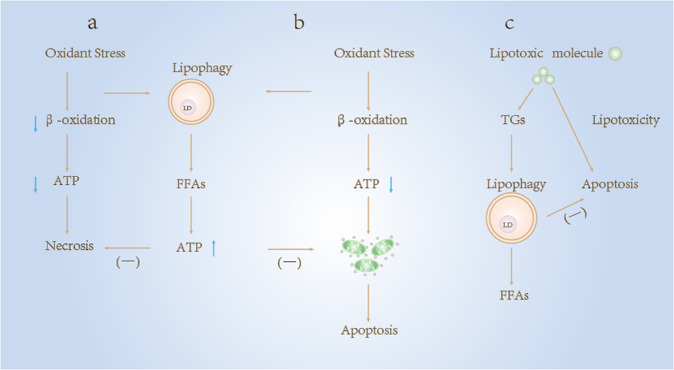
Lipophagy alters cell death responses. **a**, **b** Oxidative stress decreases the rate of mitochondrial β-oxidation, resulting in a decrease in cell ATP content. Severe reductions in ATP can lead to necrosis and cell death. Decreased levels of ATP promote mitochondrial dysfunction and trigger apoptotic cell death by affecting the release of cytochrome c. Lipophagy mediates resistance to these two forms of cell death by degrading lipid droplets into fatty acids and participating in the synthesis of ATP. **c** Lipotoxic molecules can cause apoptosis directly or by inhibiting lipophagy, which degrades lipotoxic molecules by enveloping triglycerides in lipid droplets and degrading them.

The relationship between autophagy and cell death has been explored, and many researchers speculate that the two processes are related [89]. Autophagy was previously considered a form of cell death, but in recent years, studies have gradually shown that autophagy promotes survival to some extent [90, 91]. As a selective autophagy, lipophagy is a mechanism of lipid metabolism and plays a role in resisting cell death; if lipophagy function is disrupted, lipids can become toxic and eventually trigger cell death. For example, the 22-carbon hexaenoic acid (DHA22:6n-3) in nerve growth factor differentiated pheochromocytoma (NGFDPC12) enhances autophagy by upregulating ATG7 and ATG12 and protects cells from palmitic acid (PAM)-mediated lipotoxicity (PAM-LTX) by inhibiting necrosis and apoptosis [92]. Thus, lipophagy may serve a cytoprotective function by decomposing lipids to maintain energy supply, preventing necrosis, apoptosis and ATP consumption induced by mitochondrial dysfunction, and promoting the metabolism of potentially lipotoxic molecules (Fig. 4).

Lipophagy has been shown to play a role in hepatocyte resistance of oxidative stress-induced death. It was previously found that knockout of the ATG5 gene caused hepatocytes to die from oxidative stress caused by normal endogenous nontoxic concentrations of menadione; it also caused cell death at toxic levels [93]. As mentioned above, changes in the fatty acid supply do not lead to hepatocyte damage and enlargement but are caused by autophagic damage via β-oxidation [17]. This notion implies that the key to maintaining an appropriate rate of β-oxidation lies in the FFAs produced by lipophagy; however, whether this pattern is common in the response to oxidative stress in other types of cells remains to be confirmed.

Proteins and membrane organelles controlled by mTOR complex 1 (mTORC1) upregulate autophagy [94, 95], causing the release of amino acids and lipids during prolonged nutritional deprivation. Interestingly, some of the lipids released by autophagy are reacylated into triacylglycerol and packaged like LDs [96, 97]. Knockout of ATG5 or addition of autophagy inhibitors can inhibit the response to nutritional deprivation or mTORC1 inhibition, blocking the formation of LDs, which shows that the formation of these LDs is autophagy-dependent [96, 97]. In a cell culture model of glucose starvation and hypoxia, nutritional deprivation and hypoxia-induced ROS accumulation can cause DNA damage, while CHK2-mediated autophagy can regulate cell survival under stress by removing damaged mitochondria and reducing cerebral ischemia–reperfusion injury and apoptosis in mice [98].

Mitogen-activated protein kinase phosphatase-5 (MKP-5), as an extracellular signal regulator, regulates lipid metabolism, and its expression is downregulated in pancreas and primary cells of obese mice fed a high-fat diet (HFD). The inhibition of rat islet tumor cell (Rin-m5f) autophagy mediated by pamidronic acid can be restored by the overexpression of MKP-5, which protect against palmitic acid (PA)-induced apoptosis and dysfunction. As PA is an inducer of lipotoxicity, its effect can be amplified by low expression levels of MKP-5; when MKP-5 is overexpressed, it can restore PA-mediated autophagy inhibition. However, infection of HFD-fed mouse islet cells with the recombinant adenovirus Ad-MKP-5 (expressing MPK-5) inhibited HFD-induced apoptosis. In experiments of the addition of autophagy inhibitors, overexpression of MKP-5 affected the inhibition of apoptosis, apoptosis dysfunction, inflammation, and oxidative stress via PA by affecting autophagy signaling [99–102].

### Lipophagy promotes potential lipotoxic molecular metabolism

Excessive accumulation of lipids in organs may lead to cell death; this occurs in many disease states, such as obesity and metabolic syndrome. In these instances, the cell damage is due to the toxicity of saturated FFAs. These FFAs act as a source of oxidative stress that triggers hepatocyte cell death. As the two most common liver diseases, NAFLD and alcoholic liver disease (ALD) are related to abnormal increases in liver fat [103]. In contrast, lipophagy may prevent hepatocyte damage by maintaining energy balance and metabolizing toxic lipids [104].

A study in the freshwater bony fish Pelteobagrus fulvidraco found that copper (Cu) exposure could increase the content of intracellular lipids, while zinc (Zn) activation inhibited lipid sequestration induced by Cu exposure. Both Zn and Zn + Cu treatment activated lipophagy in P. fulvidraco hepatocytes, reduced lipid accumulation, and increased the release of nonesterified fatty acids (NEFAs) [105]. This inhibition of Cu-induced lipid accumulation was confirmed by the presence of Zn-induced Beclin1 deacetylation and lipophagy, which indirectly provides evidence for the causal relationship between lipid droplet decomposition and lipophagy.

Studies have shown that autophagic activity is high in alcoholic mouse livers and ethanol-treated hepatocytes in vitro, while autophagic degradation of longevity proteins is not increased [106]. Increases in the number of autophagosomes containing LDs and mitochondria suggest that selective promotion of lipophagy and mitophagy during alcohol exposure may be the reasons why autophagy can protect against ethanol-induced fatty liver injury. In both drug-treated and gene-knockout groups, high autophagic activity led to the death of ethanol-treated hepatocytes and mouse liver cells, while rapamycin decreased alcohol-induced lipid accumulation, and autophagy inhibition aggravated steatosis [107]. However, how autophagy reduces lipid accumulation in ALD and whether this may apply to other cell types requires further investigation.

During nutritional deprivation, it is puzzling to find that cells consume energy to synthesize triglycerides, which increases the number of LDs [97]. Most of the energy supply in heart tissue comes from fatty acid oxidation, making this tissue vulnerable to acylcarnitine-induced damage. In cancer cells with high autophagic flux and continual recycling of organelles [108–110], pathogenesis may be related to LDs, acylcarnitine, and cell damage. High levels of acylcarnitine can lead to diseases of overactive fatty acid oxidation as well as myocardial ischemia [111–113]. Improvement in cardiac function is not related to diacylglycerol, triacylglycerol, or ceramide but to decreases in acylcarnitine, suggesting that acylcarnitine may play a lipotoxic role in vivo [112]. The protective effects of DGAT1 in mice with cardiomyopathy [114–116] suggest that it is more advantageous to store fatty acids in LDs in the form of triglycerides and gradually release these fatty acids through lipolysis or lipophagy than to convert these fatty acids into toxic lipids. In addition, various fatty acids can modulate the rate of autophagic activity [117], but whether this involves lipophagy remains to be confirmed. As a key function of autophagy, continuous cell quality control ensures that damaged organelles [118], lipids, and proteins can regulate turnover. In most cases, autophagy promotes cell survival, but as with certain environments, particularly cancer cell death induced by lower organisms or drugs [119, 120], autophagy may actually induce cell death in a process known as autophagy-dependent cell death (ACDC) or autosis [120, 121, 122]. However, this process has yet to be shown to involve lipophagy.

## The role of lipophagy in metabolic disorders

### Fatty liver disease

In view of the previously shown important role of lipid metabolism in liver homeostasis, impaired lipid metabolism may be an important factor in excessive lipid accumulation and steatosis in the liver, which leads to serious consequences of alcoholic and NAFLD. As mentioned above, lipophagy regulation of cell death is also an important feature of the disease. Several susceptibility conditions for nonalcoholic steatohepatitis (NASH) can damage liver autophagy, which is consistent with autophagy deficiency promoting the development of fatty liver disease. NASH is associated with steatosis and liver inflammation and injury. On a high-fat diet, the autophagy activator resveratrol can reduce liver steatosis, suggesting that the drug has the potential to treat NASH [123].

NAFLD is often caused by the accumulation of lipids in the liver, which further leads to cell damage and stress and eventually to the development of liver cirrhosis and even liver cancer. Steatosis marked by abnormal accumulation of LDs is an important early pathological feature of NAFLD. Researchers have conducted a large number of experiments to explore the effects of autophagy on NAFLD and have provided important evidence that autophagy plays a key role in preventing the progression of NAFLD. Both liver biopsies from adult subjects and liver sections from donors who had died showed that a large amount of the autophagy substrate p62/SQSTM1 [124, 125] had accumulated in the livers with severe steatosis. These findings prove that the degree of autophagy is inversely proportional to liver lipid content, and the degradation of accumulated lipids may occur through lipophagy because retroviral drugs that inhibit autophagy activity can in turn increase lipid accumulation in the liver. Similarly, carbamazepine can prevent steatosis by activating autophagy [126].

Autophagy also mediates lipid degradation in in vivo and in vitro models of acute and chronic ethanol treatment and is related to the production of reactive oxygen intermediates [107, 127]. This finding suggests that autophagy protects against oxidative stress and liver injury caused by acute and chronic ethanol treatment by degrading lipids and that removing excess lipids or generating FFAs via lipophagy to provide ATP may be the mechanism by which hepatocytes protect against oxidative stress [93].

Lipophagy mediates the fibrotic process of differentiation of hepatic stellate cells (HSCs) into myofibroblasts (MFBs). There is considerable lipid storage in the form of vitamin A in HSCs. The progression of fibrosis leads to the appearance of fibrous nodules in the liver, which destroys the structure and function of the liver. Autophagy increases under fibrotic stimulation, and inhibition of stellate cell autophagy blocks activation, but activation can be restored by FFA supplementation, suggesting that lipophagy may promote the process by providing the energy needed for activation [128]. Therefore, the inhibition or regulation of lipophagy in fibrosis may be an effective treatment.

When HSCs are activated in vivo, a significant increase in autophagic flux is observed, and double-membrane autophagosomes containing LDs are detected in vitro. Peroxisome proliferator activated receptor α (PPAR α) can induce lipophagy in vivo but may promote fibrosis [129]. Lipophagy in HSCs may be mediated by Rab25 and dependent on reactive oxygen species. SiRNA interference of Rab25 can inhibit the activation of LDs [130] by reducing HSC degradation. Knockout of ATG7 or ATG5 can inhibit lipophagy and delay the process of fibrosis in the livers of mice. Lipophagy promotes the decomposition of LDs and accelerates the activation of HSCs, which eventually leads to the progression of liver fibrosis [131]. Therefore, targeted blockade of lipophagy of HSCs should be an expected antifibrosis strategy. These findings indicate that lipophagy plays an important role in maintaining lipid levels in the liver and provide a basis for us to understand the molecular mechanism of lipid metabolism mediated by lipophagy.

### Cancer

Lipophagy has been shown to play a role in various diseases; targeted stimulation of lipophagy represents a feasible method of treating fatty liver disease, while in NAFLD, activation of autophagy/lipophagy is also being studied [48, 49]. In NAFLD models, intraperitoneal injection of carbamazepine and rapamycin can alleviate liver steatosis in mice [50]. Small molecules such as resveratrol and caffeine in addition to being shown to alter the gene expression of TFEB have been shown to reduce steatosis in the liver [51–53]. In glucose-6-phosphatase deficiency (von Gierke disease), genetically or chemically stimulating autophagy can reduce lipid accumulation in the liver and reduce steatosis [55]. Moreover, other studies have suggested that LD-mediated autophagy activation and lipophagy-driven catabolism may serve as markers of hepatic stellate cell activation and extracellular matrix deposition in fibrotic liver tissue [54]. Thus, if we can discern the molecular mechanisms of lipophagy and its regulation, we can develop methods to modulate lipid accumulation in NAFLD and prevent early fibrosis.

From the perspective of cancer, although metabolism-related cancer research has received increasing attention in the past decade, the regulation and role of LDs, and especially lipophagy in cancer, are still poorly understood [56]. Fatty acids produced by lipophagy participate as an important source of energy in β-oxidation, which cancer cells can use to continuously grow and proliferate. As a transcription factor overactivated in hepatocellular carcinoma, CCAAT enhancer binding protein a weakens tumorigenesis by activating lipophagy, an effect that can be abrogated by ATG knockdown or chloroquine treatment [57]. In another experimental model, Nogo-B, an ER-resident protein, was found to be highly expressed in both mouse and human NAFLD-associated hepatocellular carcinoma. Nogo-B interacts with ATG5 and, when overexpressed, can activate lipophagy, induce carcinogenicity, degrade TGs from LDs, and increase FFA content [60]. These effects are consistent with the role of autophagy in hepatocellular carcinoma and have been verified in our previous studies [61–63]. Therefore, it appears that lipophagy supports tumor growth to some extent. In one study, it was found that the inhibition of LAL is associated with tumorigenesis, tumor growth, and metastasis [65]. The absence of LAL leads to haematopoietic abnormalities, inhibiting the production of immature myeloid-derived suppressor cells (MDSCs) and thereby suppressing immune surveillance, which can lead to immune escape [66]. I has been confirmed in other models that MDSC deficiency induced by LAL can directly stimulate tumorigenesis and metastasis [64].

Overactivation of lipophagy is associated with poor prognosis and a poor survival rate in cancer patients [67, 132, 133], as it yields cancer cells with a plentiful source of energy as well as lipid intermediates to use in the synthesis of other biomolecules [134]. Although such studies are still in their infancy, the ones so far have identified an unrecognized role of lipophagy in cancer metabolism from an innovative perspective, providing a basis for future experiments.

P53, a common tumor suppressor protein, can directly participate in the regulation of autophagy- and fatty acid β-oxidation-related genes and activate subsequent lipid degradation [135]. P53 can induce the expression of autophagy-related genes such as ATG7 and ATG14, and UVRAG can be inhibited by pifithrin α, resulting in lipid accumulation [136, 137]. Interestingly, it has been speculated that p53 may play a unique role in the induction of lipophagy. This idea was verified in oleic acid (OA)-treated Chang hepatoma cells, in which P53 was able to abrogate the induction of lipid accumulation by OA [136, 138]. P53 is necessary for the induction of lipophagy in Chang hepatoma cells. The inhibition of p53 can downregulate the expression of LC3-II and perilipins needed for lipophagy, and thus, p53 plays a unique role as a tumor suppressor in lipophagy. The selective removal of LDs by autophagy can be damaged by the knockout of microtubule-associated protein 1s (MAP1S) in renal cells, and overexpression can increase the removal of LDs [132]. Compared with that in clear cell carcinoma, the level of MAP1S in normal renal cells is higher, and a high level of MAP1S is associated with a lower malignant degree and metastasis probability and better prognosis. These results suggest the potential role of lipid phagocytosis in cancer. Studies in HeLa cells have found a unique relationship between ATG14 and lipophagy. Lipophagy and intracellular fatty acid accumulation are promoted by ATG14 overexpression and lead to oxidative stress and apoptosis, while inhibition of ATG14 increases cell survival [139]. In androgen-sensitive prostate cancer cell lines and glioma cells [76, 139], lipophagy plays a contrasting role in providing energy to promote cell growth. However, it was also previously suggested that lipophagy, like autophagy, can play a dual role in cancer [139, 140], as in some cases, overactive lipophagy may inhibit tumor growth. This hypothesis was further supported by the fact that in triple-negative breast cancer cells, combined treatment with DHA and Delta-T3 results in a lower degree of malignancy and reduced proliferation by activating lipophagy and reducing the biogenesis of LDs [141].

It is clear that further exploration is needed to clarify the role of lipophagy in cancer. Like most other metabolic pathways, the role of lipophagy in cancer cell metabolism, growth, signal transmission, and metastasis may depend on the type and stage of the tumor as well as the characteristics of the cancer itself (Table 1). Studies have shown that lipids could be a primary alternative source of energy that supports tumor cell proliferation. The role of autophagy in the degradation of lipids stored in the form of LDs and the function of regulating intracellular lipid homeostasis are currently being elucidated to uncover the full therapeutic potential.

**Table 1:** Possible role of lipophagy in different cancer models.

Model	Gene/protein	Regulation/treatment	Effect on lipophagy	Effect	Reference
Melanoma	FAO	High-fat diet	Increase	Increases neutral lipids in LDs, promotes tumor invasiveness	[142]
Prostate cancer	SIRT1	AGG	Increase	Decreases the number of LDs, promotes the senescence of prostate cancer cells	[143]
Ovarian cancer	PKFPB3	PKF-158	Increase	Increases the decomposition of LDs, inhibits tumor growth	[144]
Cervical cancer	ATG14	Overexpression of ATG14	Increase	Increases the degradation of LDs, increases FFAs, reduces the cell survival rate	[139]
Hepatocellular carcinoma	Nogo-B	Overexpression of Nogo-B	Increase	Decreases the number of TGs, increases the number of FFAs	[60]
Hepatocellular carcinoma	Rab10	Depletion of Rab10 by siRNA	Decrease	Decreases the number of LDs	[145]
Clear cell renal cell carcinoma	ABCA1	Celastrol	Increase	Increases the degradation of LDs, inhibits the proliferation, invasion and migration of cancer cells	[146]
Breast cancer	ADRP	DHA/Delta-T3	Increase	Reduces the biogenesis of LDs, inhibits the malignant progression of tumors	[141]
Glioma	mTORC1	FAO inhibitors in combination with 2DG	Decrease	Decreases energy production and the survival of glioma cells, suppresses the progression of xenografted glioma	[147]

## Conclusion

Lipids are stored in LDs in the form of TAG and metabolized by lipolysis and lipophagy. Autophagy plays an important role in cell quality control, accumulation of misfolded proteins, and removal of damaged organelles. Although autophagy was initially considered to be a nonselective biological process, studies in yeast and other higher eukaryotic cells have shown that multiple types of selective autophagy can occur frequently in multiple cell and tissue types.

Although lipophagy is a relatively newly discovered process compared with autophagy, great progress has been made in the past few years. It has been confirmed that lipid phagocytosis plays a role in many different types of cells, indicating that this form of autophagy is a common pathway of cellular lipid metabolism. However, the mechanism by which LDs are identified as a substrate and how nutritional status regulates this process remain unclear. At present, it seems that the most important role of lipophagy is to maintain a sufficient level of β-oxidation to produce ATP. This function is thought to be related to the role of lipophagy in regulating cell death and transdifferentiation. However, there are probably other features that have not yet been described. Identifying the proteins necessary for the initiation of lipid phagocytosis may give us a better understanding of the core components that degrade LDs. Impaired lipid phagocytosis may be the basic mechanism underlying metabolic disorders, such as obesity, fatty liver, atherosclerosis and metabolic syndrome. Therefore, we also need to further explore the dynamic relationship between lipid phagocytosis processes, including microlipophagy and chaperone-mediated lipophagy, and the body as a whole. In addition, the importance of lipophagy in cancer progression has been increasingly recognized, which indicates that it is possible to find specific lipophilic receptors that can be used as targets for cancer therapy.

## Supplementary information

authorship statement

## Data Availability

All data generated or analyzed during this study are included in the main text and the Supplementary Information files.
