# Supplementary material for: The regulation, function, and role of lipophagy, a form of selective autophagy, in metabolic disorders
Source: Cell Death Dis. 2022 Feb 8;13(2):132. doi: 10.1038/s41419-022-04593-3 (PMC8825858; doi:10.1038/s41419-022-04593-3)
Supplement: Supplementary file 1 — authorship statement [file 41419_2022_4593_MOESM1_ESM.pdf]

AUTHORSHIP STATEMENT

Manuscript title: The regulation, function, and role of lipophagy, a form of selective autophagy, in metabolic Disorders.

All persons who meet authorship criteria are listed as authors, and all authors certify that they have participated sufficiently in the work to take public responsibility for the content, including participation in the concept, design, analysis, writing, or revision of the manuscript. Furthermore, each author certifies that this review article or similar review article has not been and will not be submitted to or published in any other publication before its appearance in the *Cell Death & Disease*.

Authorship contributions

Sheng Zhang, Xueqiang Peng, and Shuo Yang performed literature investigation and wrote the manuscript. Xinyu Li, Mingyao Huang, and Shibo Wei conceived the project and designed the outline. Jiaxing Liu, Guangpeng He, Hongyu Zheng, Liang Yang, Hangyu Li, and Qing Fan edited and revised the paper. All authors have reviewed the paper and all approved of the final manuscript.

Acknowledgements

All persons who have made substantial contributions to the work reported in the manuscript (e.g., technical help, writing and editing assistance, general support), but who do not meet the criteria for authorship, are named in the Acknowledgements and have given us their written permission to be named. If we have not included an Acknowledgements, then that indicates that we have not received substantial contributions from non-authors.

This statement is signed by all the authors:

Author's name (typed)	Author's signature	Date
Sheng Zhang	[illegible] Sheng Zhang	2022.1.5
Xueqiang Peng	[illegible] Xueqiang Peng	2022.1.5
Shuo Yang	[illegible] Shuo Yang	2022.1.5
Xinyu Li	[illegible] xinyu Li	2022.1.5
Mingyao Huang	[illegible] Mingyao Huang	2022.1.5
Shibo Wei	[illegible] Shibo Wei	2022.1.6
Jiaxing Liu	[illegible] Jiaxing Liu	2022.1.5
Guangpeng He	[illegible] Guangpeng He	2022.1.5

Hongyu Zheng	郑宏宇 Hongyu Zheng	2022.1.5
Liang Yang	杨亮 Liang Yang	2022.1.7
Hangyu Li	李航宇 Hangyu Li	2022.01.06
Qing Fan	范青 Qing Fan	2022.1.6

AUTHORSHIP STATEMENT

Manuscript title:

The regulation, function, and role of lipophagy, a form of selective autophagy, in metabolic Disorders.

All persons who meet authorship criteria are listed as authors, and all authors certify that they have participated sufficiently in the work to take public responsibility for the content, including participation in the concept, design, analysis, writing, or revision of the manuscript. Furthermore, each author certifies that this review article or similar review article has not been and will not be submitted to or published in any other publication before its appearance in the *Cell Death & Disease*.

Authorship contributions

Sheng Zhang, Xueqiang Peng, and Shuo Yang performed literature investigation and wrote the manuscript. Xinyu Li, Mingyao Huang, and Shibo Wei conceived the project and designed the outline. Jiaxing Liu, Guangpeng He, Hongyu Zheng, Liang Yang, Hangyu Li, and Qing Fan edited and revised the paper. All authors have reviewed the paper and all approved of the final manuscript.

Acknowledgements

All persons who have made substantial contributions to the work reported in the manuscript (e.g., technical help, writing and editing assistance, general support), but who do not meet the criteria for authorship, are named in the Acknowledgements and have given us their written permission to be named. If we have not included an Acknowledgements, then that indicates that we have not received substantial contributions from non-authors.

This statement is signed by all the authors:

Author's name (typed)	Author's signature	Date
Sheng Zhang	[illegible] Sheng Zhang	2022.1.5
Xueqiang Peng	[illegible] Xueqiang Peng	2022.1.5
Shuo Yang	[illegible] Shuo Yang	2022.1.5
Xinyu Li	[illegible] Xinyu Li	2022.1.5
Mingyao Huang	[illegible] Mingyao Huang	2022.1.5
Shibo Wei	[illegible] ~~Wei~~ Shibo Wei	2022.1.6
Jiaxing Liu	[illegible] Jiaxing Liu	2022.1.5
Guangpeng He	[illegible] Guangpeng He	2022.1.5

Hongyu Zheng	郑宏宇 Hongyu Zheng	2022.1.5
Liang Yang	杨亮 Liang Yang	2022.1.7
Hangyu Li	李航宇 Hangyu Li	2022.01.06
Qing Fan	范青 Qing Fan	2022.1.6
